# Radiation-induced gastrointestinal (GI) syndrome as a function of age

**DOI:** 10.1038/s41420-023-01298-0

**Published:** 2023-01-25

**Authors:** Hongyan Li, Herman C. Kucharavy, Carla Hajj, Liyang Zhao, Guoqiang Hua, Ryan Glass, Phillip B. Paty, Zvi Fuks, Richard Kolesnick, Karen Hubbard, Adriana Haimovitz-Friedman

**Affiliations:** 1 Department of Radiation Oncology, Memorial Sloan Kettering Cancer Center, New York, NY USA; 2 Department of Biology, The City College of New York, New York, NY 10031 USA; 3 CUNY Graduate Center, New York, NY 10016 USA; 4 Laboratory of Signal Transduction, Memorial Sloan Kettering Cancer Center, New York, NY USA; 5 Department of Surgery Memorial Sloan Kettering Cancer Center, New York, NY USA

**Keywords:** Ageing, Physiology

## Abstract

Previous studies show increased sensitivity of older mice (28–29 months) compared with young adult mice (3 months, possessing a mature immune system) to radiation-induced GI lethality. Age-dependent lethality was associated with higher levels of apoptotic stem cells in small intestinal crypts that correlated with sphingomyelinase activity, a source of pro-apoptotic ceramide. The objective of this study is to determine whether the cycling crypt base columnar cells (CBCs) in aging animals are specifically more sensitive to radiation effects than the CBCs in young adult mice, and to identify factors that contribute to increased radiosensitivity. Mortality induced by subtotal body radiation was assessed at different doses (13 Gy, 14 Gy, and 15 Gy) in young adult mice versus older mice. Each dose was evaluated for the occurrence of lethal GI syndrome. A higher death rate due to radiation-induced GI syndrome was observed in older mice as compared with young adult mice: 30 vs. 0% at 13 Gy, 90 vs. 40% at 14 Gy, and 100 vs. 60% at 15 Gy. Radiation-induced damage to crypts was determined by measuring crypt regeneration (H&E staining, Ki67 expression), CBC biomarkers (lgr5 and ascl2), premature senescence (SA-β-gal activity), and apoptosis of CBCs. At all three doses, crypt microcolony survival assays showed that the older mice had fewer regenerating crypts at 3.5 days post-radiation treatment. Furthermore, in the older animals, baseline CBCs numbers per circumference were significantly decreased, correlating with an elevated apoptotic index. Analysis of tissue damage showed an increased number of senescent CBCs per crypt circumference in older mice relative to younger mice, where the latter was not significantly affected by radiation treatment. It is concluded that enhanced sensitivity to radiation-induced GI syndrome and higher mortality in older mice can be attributed to a decreased capacity to regenerate crypts, presumably due to increased apoptosis and senescence of CBCs.

## Introduction

Gastrointestinal acute radiation syndrome (GI-ARS) (also known as radiation GI syndrome [RGS], is a major toxicity associated with abdominal irradiation. This syndrome presents with anorexia, vomiting, diarrhea, infection, and, in extreme cases, septic shock and death [1]. GI-ARS is thought to result from damage to intestinal stem cells (ISCs) residing in the crypts of Lieberkuhn, ultimately leading to the loss of the entire crypt [2–7]. Our earlier studies suggested that doses exceeding 8 Gy cause endothelial cell apoptosis, secondary to acid sphingomyelinase (ASMase) activity and ceramide generation, resulting in vascular compromise and impaired DNA damage repair (DDR) in the crypt stem cells [8]. Higher doses of radiation, via this additional mechanism, ultimately lead to decreased crypts and increased mortality from GI-ARS.

More recently, we showed that the application of an anti-ceramide antibody or 6B5 scFv to the GI endothelial cell surface at 24 h post-radiation had a neutralizing effect that significantly decreased ongoing microvascular [6] apoptosis, and resulted in increased ISC regeneration, crypt survival, and reduced mortality from GI-ARS [6, 9, 10]. In those studies, we identified a previously unrecognized vascular pathophysiology developing 24 h post-radiation that is responsive to ceramide. While ISC preservation is a DDR-independent process, their increase, crypt survival, and reduced animal mortality are dependent on microvascular function.

There is definitive evidence that cycling CBCs constitute an important component of the ISC compartment that supports crypt regeneration post-radiation. These cycling cells are a small ISC population that is most often located between Paneth cells at positions +1 and −4 from the crypt base [11, 12]. CBCs are characterized by high-level expression of the Wnt target gene *lgr5* (also known as Gpr49) [12]. Lineage-tracing experiments showed that a single Lgr5-positive cell generates all mouse intestinal terminally differentiated epithelial lineages over a 1-year period [12], and that a single-sorted Lgr5+ stem cell is capable of generating ever-expanding crypt/villus organoids in vitro, in which all differentiated intestinal mucosa cell lineages are present [13]. We previously showed [6] that the CBC ISC, similar to the quiescent hematopoietic stem cells (HSC) and bulge stem cells (BSC), exhibits DNA repair-mediated radiation resistance. These normal adult tissues stem cell populations, therefore, display increased DNA repair to survive genotoxic insults [14, 15].

Other studies showed that older animals display increased numbers of apoptotic stem cells in small intestinal crypts following 1–8 Gy gamma irradiation [16]. Consistent with this finding, sphingomyelinase activity, the source of pro-apoptotic ceramide, also increased with age [17]. In addition, aged ISCs exhibit decreased expression of p53 [16]. As an expression of p53 in these cells would protect them from apoptosis, down-regulation of p53 likely plays an important role in the apoptotic response to gamma radiation by small intestinal crypts [18].

As many patients diagnosed with cancer often receive radiation therapy (RT) at advanced ages, it becomes important to understand how age affects sensitivity to RT-induced GI-ARS. Epidemiological data reveal that most abdominal and GI cancers are diagnosed at the median ages of 60–72 years (SEER 2006 https://seer.cancer.gov/). However, laboratory research is frequently conducted on 8–12-week-old mice, equivalent to 8-year-old humans. Our experiments utilize 3 months old mice, equivalent to 20 years-old humans, as young adult mice, as well as 29 months- old mice, equivalent to 70–80 years-old humans, to understand changes occuring due to aging in the small intestine and yield results more applicable to the population receiving abdominal irradiation. Since a number of changes may occur with age in stem cells, our study focuses on the response of the small intestine stem cell CBCs to irradiation as relevant to the cancer patient population.

## Results

### Aged mice are more sensitive to radiation-induced GI toxicity

**Fig. 1:**
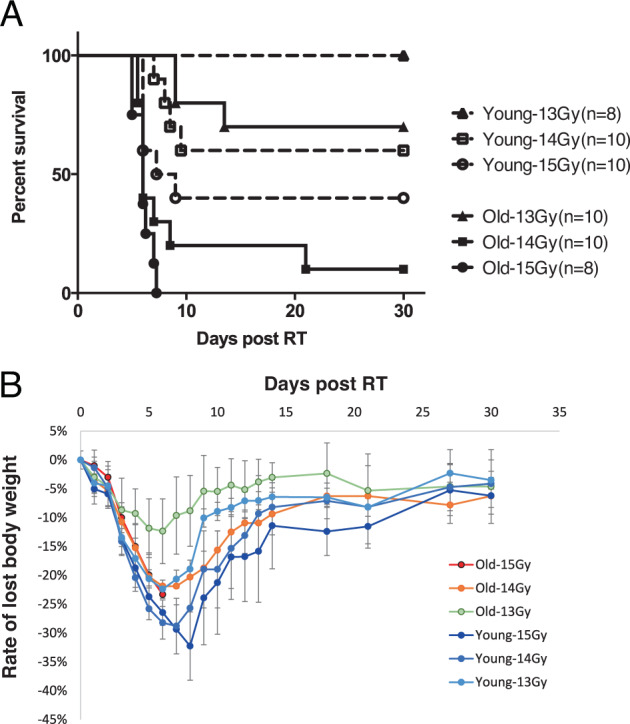
Survival curves of 3 months young mice (Y) and 29 months old mice (O) post subtotal irradiation of 13 Gy, 14 Gy, and 15 Gy. **A** Percent of the survival curve. Numbers in parentheses indicate animal number/group. **B** Rate of lost body weight curve.

We first examined the survival of young adult (3 months old) and old (28 months old) mice following exposure to escalating doses of sub-lethal radiation at 13–15 Gy (Fig. 1A). At each dose tested, younger mice tolerated radiation exposure better than the older mice. Older animals succumbed to radiation toxicity at 13 Gy with 30% mortality within 30 days, 90% mortality within 30 days at 14 Gy, and 100% mortality at 8 days at 15 Gy. There was no death among the young adult mice exposed to a sub-lethal dose of 13 Gy, but 40% succumbed to 14 Gy after 10 days (Fig. 1A), while 15 Gy induced almost 60% death in this group within 30 days. Deaths from radiation in older mice occurred within 5–8 days of treatment, and this time course is consistent with our previous results of radiation-induced GI syndrome lethality [5, 6]. In contrast, deaths in younger mice were delayed by 1–2 days relative to the older animals. Although the rate of weight loss was higher in young adult mice, it was temporary, and these mice recovered their original weight within 8–14 days post-radiation exposure (Fig. 1B). In the older animals, only those exposed to 13 Gy recovered the weight lost, which correlated with their survival at this dose (Fig. 1A, B).

### Decreased regenerating crypts in aged mice

**Fig. 2:**
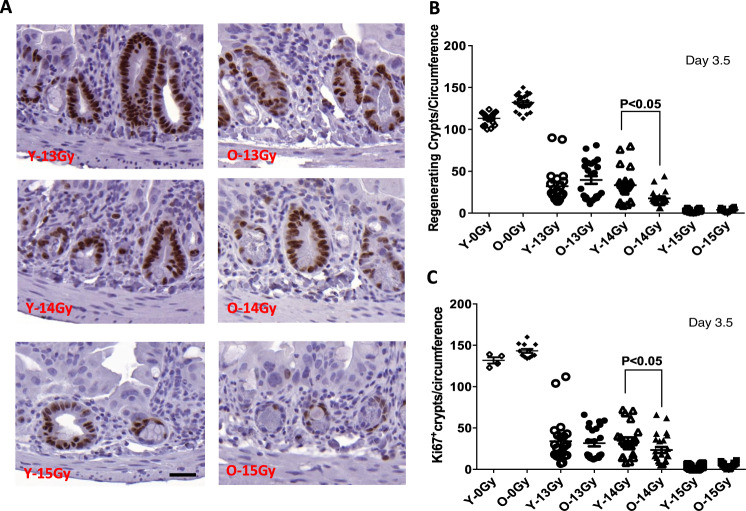
Regenerating crypts per jejunum circumference at 3.5 days post subtotal RT. The sections of proximal jejunum were obtained from animals sacrificed at 3.5 days post-radiation. **A** Representative images of regenerating crypt 3.5 days post subtotal RT. **B** Quantitation of regenerating crypt numbers. A regenerating crypt was defined as a crypt containing at least 1 Paneth cell, over 10 non-Paneth cells and 1 lumen; appearing intensely stained body in HE-section. **C** Quantitation of regenerating crypt number was scored by counting Ki67-positive crypts. Data are presented as means ± SD. *N* = 3 mice/group, 10 circumferences per mouse. *P < 0.05 Y-14Gy vs O-14 Gy, Student’s *t*-test. Scale bar 20 μm.

Next, we examined jejunal circumferences for regenerating crypts at 3.5 days post-irradiation using H&E and Ki67 stained sections as standardized in the Withers and Elkind Clonogenic Survival Assay [19]. At a dose of 13 Gy, which failed to induce GI death in the young adult group, the percentage of regenerating crypts, was similar in young adult and old mice (28.2 vs 30.0%) as compared to age-matched controls. At 14 Gy, which showed GI-mediated effects, a difference became apparent. At this dose, significantly fewer regenerating crypts were found in older mice: 28.5% in young mice vs. 14.9% in older mice, (*p* < 0.001). At 15 Gy, regenerating crypts in both groups were quite low: 2.0% in young vs 4.9% in old (*p* < 0.01). A similar trend was observed for the proliferating activity of these crypts, as measured by Ki67 staining (Fig. 2C). Equivalent proportions of proliferating crypts were seen in young adult vs. old mice after 13 Gy (25.1 vs 22.2%). At 14 Gy, a difference was observed between young adult vs. old mice (23.6 vs 16.3%, *p* < 0.05), indicative of more regeneration within young adult crypts. Fewer proliferating crypts were observed in animals exposed to 15 Gy: 1.8% in young mice vs. 3.6% in old mice (*p* < 0.05; Fig. 2). These results indicate that upon exposure to sub-lethal doses of radiation (14 Gy, 15 Gy), the two groups of mice display a significant difference in their ability to repair crypt damage.

### Aged mice lose more CBCs

**Fig. 3:**
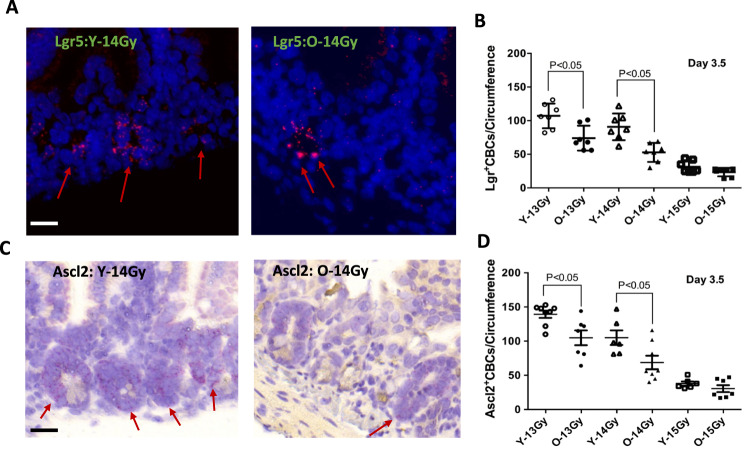
Quantification of CBC per jejunum circumference at 3.5 days post subtotal RT. Red arrows indicate the positive staining of Lgr5 in-situ hybridization staining fluorescence (Upper left & center, 40X magnification) andAscl2 in-situ hybridization bright field (Lower left & center, 40X magnification). **A** Representative images of data of Lgr5 positive CBC per circumference young (upper left panel) and older mice (upper center panel). **B** Quantification of Lgr5 positive per jejuna circumference. Lgr5 positive CBC are presented as means ± SD. *N* = 3 mice/group, 10 circumferences per mouse. **C** Representative images of Ascl2 positive CBC per jejuna circumference young (bottom left panel) and older positive Ascl2 per circumference (bottom center panel). **D** Quantification of Ascl2 positive CBC per jejuna circumference. *P < 0.05 Y-13Gy vs O-13 Gy and Y-14 Gy vs O-14 Gy, Student’s *t*-test.Scale bar 20 μm.

To better assess the response to radiation, we next examined CBC survival in response to radiation using two CBC markers: Lgr5 and Ascl2 (Fig. 3). The total Lgr5-positive cells per circumference were 394 ± 35 (average ± s.d.) in 3-month-old adults and 412 ± 64 in 29-month- old mice (Fig. 3A, B). Upon examination of the crypts for Lgr5 expression at 3.5 days post-IR, younger mice demonstrated more Lgr5-positive CBC per circumference relative to older mice, after exposure to either 13 Gy or 14 Gy: 107.1 vs. 75 (*p* ≤ 0.004), and 90.9 vs. 52.9 (*p* = 0.03), respectively. Younger mice continued to show higher Lgr5-positive CBC treated with 15 Gy, although the difference (31.4 vs. 23.4) was no longer significant. Similar results were obtained when assessing the additional CBC marker, Ascl2 (Fig. 3D). Comparably, young mice continued to show more Ascl2 positive cells per circumference than older animals after treatment with 13 Gy: 137.3 vs. 104.9, (*p* = 0.01), and after 14 Gy: 106.7 vs. 68.88 (*p* = 0.03). Similar to results obtained with Lgr5, this difference was lost after 15 Gy (30 vs 30.6).

### CBC apoptosis

**Fig. 4:**
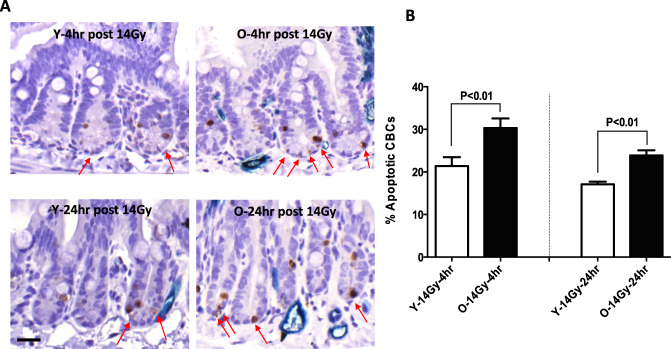
Apoptotic CBCs in response in young vs old mice at 4 h and 24 h post-RT. **A** The sections of proximal jejunum were obtained from young adult and old mice sacrificed at 4h (upper left and center panels) and 24 h post 14 Gy irradiation(bottom left and center panels). The apoptosis was detected with cleaved caspase-3 by IHC staining. Apoptosis Index = positive apoptotic CBC/total CBC per jejuna circumference x 100%. Red arrows indicate apoptotic CBCs. **B** Quantification of percent apoptotic CBC per jejuna circumference. Data are presented as means ± SD. *N* = 3 mice/group, 10 circumferences per mouse. **P < 0.01 Y-14 Gy-4 h vs O-14 Gy-4 h and Y-14 Gy-24 h vs O-14 Gy-24 h, Student’s *t*-test. Scale bar 20 μm.

A greater apoptotic index of CBCs was seen in older mice after 13 Gy and 14 Gy. As shown in Fig. 4, we compared the number of stem cells undergoing apoptosis in young and old mice in response to 14 Gy. There was an increase in the number of apoptotic CBCs in response to this dose in both groups, but there were significantly more apoptotic cells present in the older mice (Fig. 4A, B). The apoptotic indices of CBCs at 4 h post-irradiation were 21.4 ± 6.7% in young adults and 30.3 ± 5.5% in the old mice (*p* = 0.009). At 24 h post-14Gy, the values were 17.1 ± 1.9% and 23.9 ± 3.9%, respectively (*p* = 0.0001).

### CBC senescence

**Fig. 5:**
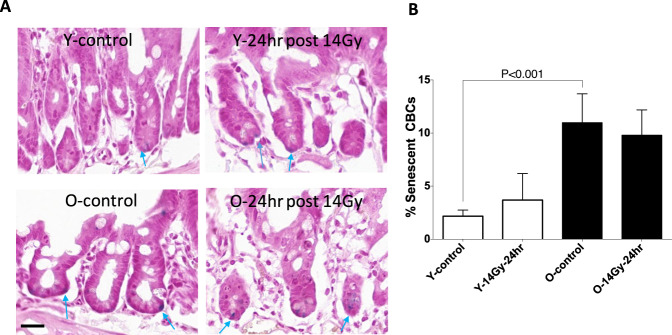
The sections of proximal jejunum were obtained from young adult and old mice sacrificed at 24 h post 14 Gy irradiation. **A** The senescence-associated β-gal (SA-β-Gal) staining was detected with a commercial kit. Senescent index = positive β-gal CBC/total CBC per jejuna circumference × 100%. Blue arrows indicate senescent CBCs. **B** Quantification of percent senescent CBC per jejuna circumference. Data were presented as means ± SD. *N* = 3 mice/group, 10 circumferences per mouse. ***P < 0.001 Y-control vs O-control, Student’s *t*-test. Scale bar 20 µm.

Radiation has been reported to induce senescence [20]. Therefore, we measured irradiation-induced CBC senescence at 24 h after subtotal radiation. A significant increase in the number of senescent CBCs was observed in older mice vs. young mice at baseline, but irradiation had no further effect on this parameter in either younger or older mice (Fig. 5).

### Paneth cell proliferation

**Fig. 6:**
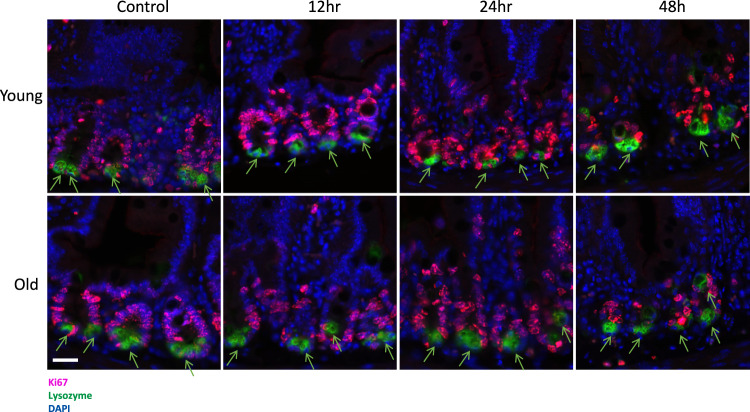
The sections of proximal jejunum were obtained from young adult and old mice sacrificed at 12, 24, and 48 h post 14 Gy irradiation. Paneth cell proliferation was assessed by IHF double staining of nuclear protein Ki67 (red) and Paneth cell marker lysozyme (green). Green arrows indicate lysozyme staining. Representative images containing 4 to 6 Paneth cells were shown. Scale bar 20 µm.

Subsequently, we confirmed that the increase in CBC number in older mice did not result from the differential division of Paneth cells (Fig. 6) [21]. Paneth cell proliferation was assessed from the sections of proximal jejunum obtained from young adult and old mice sacrificed at 12, 24, and 48 h post-14Gy. Two to three mice were used in each group, with 12 circumferences analyzed per mouse. By colocalizing Ki67 (red) and the Paneth cell marker lysozyme (green) as a measure of proliferation, we determined that there was no proliferation of Paneth cells as indicated by the absence of colocalization of the two markers (Fig. 6). These results indicate that the significant increase in the number of senescent CBCs observed in the old mice is likely to be a consequence of age.

## Discussion

Our data demonstrate age-related changes in sensitivity to radiation-induced GI toxicity. At doses of radiation known to cause the GI syndrome, older mice showed fewer surviving crypts and CBCs at 3.5 days post-radiation, as well as reduced survival, compared with the young adult mice. Consistent with these findings, a greater degree of apoptosis was observed in the CBC population in older mice both at 4 h and at 24 h post-radiation, after DNA damage repair (DDR) was completed [9]. These results are compatible with Potten’s findings of fewer surviving crypts in older mice at doses above 13Gy [16]. It was suggested that the altered regenerative potential of these cells may be mediated by a reduced capacity to mount a regenerative response, likely in part due to altered p53 and p21 expression. It was also proposed that aged crypts may recruit other cells into the clonogenic compartment, which is consistent with several reports of expanded proliferative zones in aged colonic crypts [22–24]. As these recruited cells may be less efficient clonogens and a larger number would need to be recruited, this hypothesis is consistent with the reported growth delay.

Several factors may be responsible for the increased sensitivity of older mice to radiation-mediated GI toxicity. We previously demonstrated that endothelial apoptosis is the initiating lesion for GI syndrome lethality [5], and also demonstrated the requirement for ASMase activity and ceramide generation for the initiation of endothelial apoptosis [8, 25, 26]. Additionally, increased ASMase and ceramide levels were seen in older mice versus their younger counterparts [17]. Taken together, the increase in endothelial apoptosis is likely to be one of the contributing factors to radiosensitivity acquired with age. Though few apoptotic endothelial cells were detected after 14 Gy irradiation, an increase in endothelial apoptosis was found after 16 Gy irradiation in both groups, with significantly more apoptotic cells present in the irradiated older mice. Thus, irradiation-induced endothelial apoptosis may contribute to the GI sensitivity of older mice.

Since we observed no increase in the proliferation of Paneth cells in younger mice (Fig. 6), regeneration of crypts in this group did not result from Paneth cells or their lineage-committed precursors that have the capacity to dedifferentiate following irradiation [21].

Senescence may occur prematurely in response to various stress stimuli such as oxidative stress or DNA damage [27]. Stem cells in aged tissues experience long-term exposure to genotoxic assaults, from both endogenous and exogenous sources. Elevated levels of damaged DNA in aged stem cells could result from an accumulation of damage over time, an increase in the rate of damage, a decrease in the rate of repair in response to DNA damage, or a combination of these factors. Accumulation of DNA damage in stem cells may trigger the production of defective progeny, stem cell senescence or neoplastic transformation, leading to age-dependent loss of organ function and homeostasis [28]. In our study, we did not observe the senescence of CBCs in response to irradiation either in young or older mice. However, an increase of senescent CBCs was found in untreated, control-old mice, consistent with an impaired ability to recruit additional cells as clonogens. Increased senescence would predict decreased numbers of surviving CBCs and an inability to recruit more CBCs, resulting in decreased ability to regenerate the crypt and thereby causing enhanced sensitivity to radiation.

We propose that during the evolution of parenchymal tissue damage after radiation, ASMase/ceramide-dependent endothelial apoptosis likely represents a feed-forward process, which, once disrupted, even for a brief period, initiates a previously unknown tissue reparative process as shown recently with Sildenafil in erectile dysfunction (ED) and in cardiovascular disease (CVD) [29] https://www.biorxiv.org/content/10.1101/2021.04.08.438992v1. This prolonged protection following treatment is reminiscent of the impact of Lucentis, a fab fragment of bevacizumab, which, despite a short half-life (in days), provides a durable therapeutic outcome (for months), mitigating microvascular damage and preventing further pathology in diabetic macular edema after only a few injections [30].

Detailed analysis of loss of CBCs and their regeneration reveals that approximately one-third die by apoptosis during the growth arrest DNA reparative phase during day 1 after potentially lethal irradiation [7, 31], while two-thirds die during the rapid regenerative phase occurring at 24–48 h after irradiation. Indeed, we observed similar results in this study. These effects precede crypt loss, which occurs at 48–72 h after irradiation, and crypt regeneration, which peaks at 84 h after irradiation [6, 31]. Whether anti-ceramide 6B5 scFv mitigation of endothelial cell death delivered at 24 h post-irradiation protects ISCs from mitotic death, secondary injury from ongoing tissue damage, or enhances their regeneration is a topic of an ongoing investigation in our laboratory.

In summary, our study has further demonstrated age-related changes in the sensitivity of mice to radiation-induced GI-ARS. Stem cell populations in adult specialized tissues, whether quiescent or cycling, are radioresistant owing to the proficient use of DDR pathways. Additional experiments will be needed to elucidate the mechanisms underlying CBC dysfunction that emerge during the aging process. As the incidence of cancer increases with age, these studies should be extended to include animals with various cancer burdens to explore the role of age and cancer on sensitivity to radiation-induced GI-ARS.

## Materials and methods

### Mice

C57Bl/6 male mice, 3 (young) and 29 months old (old), were purchased from NIA/NIH. Mice were housed at the animal core facility of Memorial Sloan-Kettering Cancer Center. This facility is approved by the American Association for Accreditation of Laboratory Animal Care and is maintained in accordance with the regulations and standards of the United States Department of Agriculture and the Department of Health and Human Services, National Institutes of Health. Protocols for conducting animal experiments were approved by the Memorial Sloan-Kettering Cancer Center Research Animal Resource Center. At the end of the experiments, mice were sacrificed by hypercapnia asphyxiation and 2.5 cm segments of proximal jejunum (2 cm distal to the ligament of Trietz) were obtained. These intestinal tissues were fixed by overnight incubation in 4% neutral buffered formaldehyde. Fixed tissues were embedded in paraffin blocks and sections of the full organ circumference (5-μm-thick) were obtained by microtomy and adhered to polylysine-treated slides for H&E, IHC, and IF staining.

### Radiation delivery

Sub-lethal radiation was delivered with a Therapax DXT300 X-ray irradiator (Pantak, Inc., East Haven, CT) using 2.0 mm Al filtration (300 KVp) at a dose rate of 1.18 Gy/min. Lead shields were used to protect the heads and front legs of the mice while exposing their GI tracts and hind legs.

### Survival of mice

Actuarial survival was calculated by the product limit Kaplan–Meier method and the statistical significance of differences in survival were calculated by the Mantel log-rank test. Causes of death were evaluated by autopsies, performed within 60 min of animal death or when terminally sick animals showing an agonal breathing pattern were killed by hypercapnia asphyxiation.

### mRNA in situ hybridizations

Two distinct markers, Lgr5 [12] and Ascl2 [32], were used to determine CBCs by in situ hybridization. Commercial kit QuantiGene ViewRNA ISH Tissue 1-Plex Assay (Affymetrix, CA) was used to stain CBCs according to the user manual. Briefly, the slides were deparaffinized, pretreated, hybridized with target probe Lgr5/Ascl2 (Affymetrix) and label probe step by step, and then applied fast red substrate, followed by counterstaining with hematoxylin (Vector Laboratories, Inc., CA) and DAPI (Sigma).

### Immunohistochemistry (IHC)/immunofluorescence (IF)

The sections of proximal jejunum were obtained from young and old mice sacrificed at 4 and 24 h post 14 Gy irradiation. The 5-μm-thick slides were deparaffinized, re-hydrated, and retrieved with antigen unmasking solution H-3300 (Vector, CA) in a steamer for 30 min. After 30 min of blocking with 10% normal serum from the same species, primary antibodies or corresponding control isotype IgG was applied to the slides and incubated overnight at 4 °C. Then, after incubation with biotinylated secondary antibodies and a VECTASTAIN ABC kit (Vector labs, PK-4000), the slides were developed with DAB detection and counterstained with hematoxylin (Vector, CA). For fluorescence staining, the slides were incubated with fluorochrome-conjugated secondary antibodies in the dark and counterstained with DAPI.

Ki67 antibody (BD Pharmingen, cat# 550609) was used at 2.5 μg/ml, coupled with biotinylated goat anti-mouse IgG (Vector, cat#BA-9200, 1:250). Primary cleaved caspase-3 (Asp175) (Cell Signaling, Ca#9661) was incubated at 1:100 dilution, followed by goat anti-rabbit IgG (Vector, cat #BA-1000, 1:1000 dilution).

### Senescence-associated β-galactosidase (SA-β-Gal)

Senescent CBCs were identified by the biomarker senescence-associated β-galactosidase (SA-β- gal) activity [33]. Intestinal tissues were immediately flushed and fixed for 2 h in a 20-fold volume of ice-cold fixative (1% formaldehyde, 0.2% glutaraldehyde, and 0.02% NP40 in PBS) at 4 °C on a rolling platform, cryopreserved in 30% sucrose for 2 h embedded in O.C.T. (TissueTek) and stored at −80 °C. Cryosections (4 μm thick) were used for SA-β-Gal staining according to the instructions of the Senescence Detection Kit (MBL, Cat# JM-K320-250, MA), and counterstained with Nuclear Fast Red (Vector).

### Quantification of regenerating crypts, CBCs, and Paneth cell proliferation

Regenerating crypts and CBC numbers were measured from the sections of proximal jejunum obtained from young adult and old mice sacrificed at 3.5 days after irradiation. A regenerating crypt was defined as a crypt containing at least one Paneth cell, >10 non-Paneth cells, and an intact lumen, appearing intensely basophilic in H&E sections [6]. A regenerating crypt was also detected by IHC staining of Ki67, which was defined as containing at least three Ki67-positive cells. Two to three mice were used in each group, with 12 circumferences analyzed per mouse. CBC numbers were obtained from intestinal tissue specimens obtained 3.5 days after irradiation by in situ hybridization of Lgr5 and Ascl2. A positive cell was defined as having 3 positive Lgr5 or Ascl2 dots. Cells were quantified from at least 10 circumferences in two to three mice per group. Paneth cell proliferation was assessed from the sections of proximal jejunum obtained from young and old mouse populations that had been sacrificed at 4 and 24 h post 14 Gy irradiation by the criteria mentioned above. Paneth cell proliferation was defined by the colocalization of Ki67 (red) and lysozyme (green).

### Quantification of apoptotic CBCs

Apoptotic CBCs were defined as a positive cleaved caspase-3 stain by IHC. Apoptosis index = total apoptotic CBCs/total CBCs per circumference × 100%. Data were obtained from 10 circumferences of the small intestine of three mice per group. Senescent CBC staining was performed on the cryosections of jejunum obtained 24 h after 14 Gy. Senescent index = positive β-gal CBCs/total CBCs per circumference × 100%. Data were obtained from 6 circumferences of the small intestine of three mice per group.

### Statistics analysis

Statistical analysis was performed using the Student’s *t*-test.

## Data Availability

All data were available in the main text.
