# Correction: Radiation-induced gastrointestinal (GI) syndrome as a function of age

**DOI:** 10.1038/s41420-023-01374-5

**Published:** 2023-03-20

**Authors:** Hongyan Li, Herman C. Kucharavy, Carla Hajj, Liyang Zhao, Guoqiang Hua, Ryan Glass, Phillip B. Paty, Zvi Fuks, Richard Kolesnick, Karen Hubbard, Adriana Haimovitz-Friedman

**Affiliations:** 1 Department of Radiation Oncology, Memorial Sloan Kettering Cancer Center, New York, NY USA; 2 Department of Biology, The City College of New York, New York, NY 10031 USA; 3 CUNY Graduate Center, New York, NY 10016 USA; 4 Laboratory of Signal Transduction, Memorial Sloan Kettering Cancer Center, New York, NY USA; 5 Department of Surgery Memorial Sloan Kettering Cancer Center, New York, NY USA

**Keywords:** Ageing, Physiology

Correction to: *Cell Death Discovery* 10.1038/s41420-023-01298-0, published online 25 January 2023

The original version of this article contained a mistake. Due to a typesetting error in the “Introduction” section the references are out of order. We apologize for the error. The original article has been corrected.

